# Long Non-Coding RNAs as Surrogate Indicators for Chemical Stress Responses in Human-Induced Pluripotent Stem Cells

**DOI:** 10.1371/journal.pone.0106282

**Published:** 2014-08-29

**Authors:** Hidenori Tani, Yasuko Onuma, Yuzuru Ito, Masaki Torimura

**Affiliations:** 1 Research Institute for Environmental Management Technology, National Institute of Advanced Industrial Science and Technology (AIST), 16-1, Onogawa, Tsukuba, Ibaraki, Japan; 2 Research Center for Stem Cell Engineering, National Institute of Advanced Industrial Science and Technology (AIST), Tsukuba Central 4, 1-1-1 Higashi, Tsukuba, Ibaraki, Japan; University of Minnesota Medical School, United States of America

## Abstract

In this study, we focused on two biological products as ideal tools for toxicological assessment: long non-coding RNAs (lncRNAs) and human-induced pluripotent stem cells (hiPSCs). lncRNAs are an important class of pervasive non-protein-coding transcripts involved in the molecular mechanisms associated with responses to cellular stresses. hiPSCs possess the capabilities of self-renewal and differentiation into multiple cell types, and they are free of the ethical issues associated with human embryonic stem cells. Here, we identified six novel lncRNAs (CDKN2B-AS1, MIR22HG, GABPB1-AS1, FLJ33630, LINC00152, and LINC0541471_v2) that respond to model chemical stresses (cycloheximide, hydrogen peroxide, cadmium, or arsenic) in hiPSCs. Our results indicated that the lncRNAs responded to general and specific chemical stresses. Compared with typical mRNAs such as p53-related mRNAs, the lncRNAs highly and rapidly responded to chemical stresses. We propose that these lncRNAs have the potential to be surrogate indicators of chemical stress responses in hiPSCs.

## Introduction

Abiotic and biotic stresses in human cells are often a result of sudden and/or frequent changes in environmental factors. The molecular response to stress involves elaborate modulation of gene expression with homeostatic, ecological, and evolutionary importance. Cellular stress responses are highly conserved cellular responses to environmental changes with transient reprogramming of transcriptional, translational, and post-translational activities [1]. Such changes can damage macromolecules, including DNA, RNA, proteins, and lipids, which require replenishment.

Long non-coding RNAs (lncRNAs) are an important class of pervasive non-protein-coding transcripts involved in various biological functions [2]–[4]. The majority of lncRNAs are transcribed by RNA polymerase II (Pol II), as evidenced by Pol II occupancy, 5′ caps, histone modifications associated with Pol II transcriptional elongation, and polyadenylation [5]. There is increasing evidence of lncRNA involvement in diverse biological processes such as signals, decoys, guides, and scaffolds [6]. lncRNAs show cell type-specific expression and respond to diverse stimuli, suggesting that their expression is under considerable transcriptional control. Furthermore, lncRNAs can serve as molecular signals because transcription of individual lncRNAs occurs at a very specific time and place to integrate developmental cues, interpret cellular context, and respond to diverse stimuli [7]. lncRNA-p21 is induced by DNA damage caused by doxorubicin, and plays a key regulatory role in the p53 transcriptional response [8]. This lncRNA represses p53-regulated genes through binding to heterogeneous nuclear ribonucleoprotein K and modulating its localization, which is necessary for the p53-dependent apoptotic response to DNA damage. The lncRNA PANDA is also induced by DNA damage in a p53-dependent manner [9]. PANDA interacts with the transcription factor NF-YA to limit the expression of proapoptotic genes and enables cell-cycle arrest. Depletion of PANDA markedly sensitizes human fibroblasts to apoptosis by doxorubicin. Moreover, numerous lncRNAs, including MAGI2 antisense RNA 3 and LOC730101, are induced by DNA damage caused by doxorubicin or mitomycin C [10]. Growth arrest-specific 5 (GAS5) lncRNA is induced by serum starvation, resulting in the arrest of cellular growth. GAS5 functions as a starvation- or growth arrest-linked riborepressor for the glucocorticoid receptor (GR) by binding to the DNA-binding domain of the GR [11], [12]. These previous reports suggest that lncRNAs may act as key regulatory nodes in multiple transcriptional pathways, serving as both a signal and convenient means of tracking the transcriptional activity of promoters in response to stimuli.

To monitor cellular stress responses, the cell types are critical. Immortalized cell lines are genetically altered, typically aneuploid, and may exhibit clinically irrelevant toxic responses to compounds. Isolated cells from animal tissues lose their in vivo phenotype, can exhibit high variability among isolations, and can often only be expanded by dedifferentiation [13]. Many of these limitations can be overcome using human-induced pluripotent stem cells (hiPSCs) [14]–[17]. hiPSCs have two important capabilities: (1) pluripotency, the ability to differentiate into a variety of cells, and (2) self-renewal, the ability to undergo numerous cycles of cell division while maintaining their cellular identity. In addition, hiPSCs are free of the ethical issues associated with human embryonic stem cells. These characteristics make hiPSCs a promising choice for not only regenerative medicine research but also monitoring of environmental stresses [18].

In this study, we hypothesized that certain lncRNAs in hiPSCs highly and rapidly respond to environmental stresses. Thus, we attempted to identify novel lncRNAs that respond to chemical stresses in hiPSCs. We found six lncRNAs that accumulate in response to model chemical stresses. Our results suggest that distinct sets of lncRNAs play roles in cellular defense mechanisms against specific stresses, and that particular lncRNAs have the potential to be surrogate indicators for cellular stress responses in hiPSCs.

## Materials and Methods

### Cell culture

hiPSC line 201B7 was provided by the RIKEN BRC in Japan. The hiPSC is derived from human dermal fibroblasts, which is facial dermis of 36-year old Caucasian female [14]. hiPSC line 201B7 was maintained in Primate ES Cell Medium (ReproCELL) supplemented with 4 ng/mL Recombinant Human FGF basic (146 aa), CF (R&D Systems), and penicillin-streptomycin (Life Technologies) on mitomycin C-treated mouse embryonic fibroblasts (SNL 76/7; DS Pharma Biomedical) as feeder cells at 37°C in a humidified incubator with 5% CO_2_. For chemical stress treatments, hiPSCs were cultured in mTeSR1 Medium Kit (Stem Cell Technologies) on BD Matrigel hESC-qualified matrix (BD) without feeder cells.

### Chemical stress treatments

hiPSCs were treated with cycloheximide (final concentrations of 1, 10, or 100 µM; Biovision), hydrogen peroxide (1, 10, or 100 µM; Wako), Cadmium Standard Solution (Cd(NO_3_)_2_, 1 µM; Wako), or Arsenic Standard Stock Solution (As2O_3_, 100 nM; Wako), and then harvested at the indicated times after treatments. Cycloheximide, cadmium standard solution, and arsenic standard stock solution were diluted in dimethyl sulfoxide. Hydrogen peroxide was diluted in diethylpyrocarbonate-treated water.

### Reverse transcription-quantitative real-time polymerase chain reaction (RT-qPCR)

Total RNA was extracted from cells with RNAiso Plus (TaKaRa) according to the manufacturer's instructions. The isolated RNA was reverse transcribed into cDNA using PrimeScript RT Master Mix (Perfect Real Time; TaKaRa). The resulting cDNA was amplified using the primer sets listed in Table 1 with glyceraldehyde-3-phosphate dehydrogenase (GAPDH) mRNA levels used for normalization. Relative RNA quantities were the treated values normalized to untreated values. THUNDERBIRD SYBR qPCR mix (Toyobo) was used according to the manufacturer's instructions. RT-qPCR analysis was performed using a MyiQ2 (BIO-RAD).

**Table 1:** Primer pairs for RT-qPCR.

Name	Sence sequece (5′-3′)	Antisense sequece (5′-3′)
GAPDH	GCACCGTCAAGGCTGAGAAC	TGGTGAAGACGCCAGTGGA
POU5F1	ATCAAGCAGCGACTATGCAC	CCAGAGGAAAGGACACTGGT
SOX2	TACAGCATGTCCTACTCGCAG	GAGGAAGAGGTAACCACAGGG
NANOG	CCCCAGCCTTTACTCTTCCTA	CCAGGTTGAATTGTTCCAGGTC
TP53	CATGAGCGCTGCTCAGATAG	ACACGCAAATTTCCTTCCAC
CDKN1A	CTGGTACCCTCCTGGCTCTT	CCCAGTGCAGGTCAGAGG
TP53I3	AATGCTTTCACGGAGCAAATTC	TTCGGTCACTGGGTAGATTCT
BBC3	GACCTCAACGCACAGTACGAG	AGGAGTCCCATGATGAGATTGT
CDKN2B-AS1	TTGTTAGAAACCAGGCTGCAC	TTCTCTCTTTCTGTGGTTTCTCAAT
HOTAIR	CAGTGGGGAACTCTGACTCG	GTGCCTGGTGCTCTCTTACC
TUG1	CCAGACCCTCAGTGCAAACT	AATCAGGAGGCACAGGACA
GAS5	CTTGCCTGGACCAGCTTAAT	CAAGCCGACTCTCCATACCT
MIR22HG	CGGACGCAGTGATTTGCT	GCTTTAGCTGGGTCAGGACA
FLJ43663	GGGATAATTTGCCATCTGGA	CCGTTTCTTCCATTTTCCTCT
LOC100216545	CAGCCAAAATCTGGCCTACT	GTCCAGCAATCATTTTCTCGT
LINC00667	AGTTTGCGCCTTTTGGTCT	GGCCATGTGCAAAGGATTT
HCG18	CTGCCTTCTAGGGGCTCACT	ACATGCTCCACCAACTTTCA
LOC550112	CTGAAATCAGAGCCTGCACA	TCAAGGACCTGGAAATGACC
LINC00662	GTTTGATTTCTCGCAGACCAG	GCGAGGTCTAACCCAGGTG
GABPB1-AS1	AGGGAAAGAAAATATGCCATTTCTA	ATCATTCCGCCGCTTTCT
FLJ33630	GCAATTATCACGGGAAACCTAT	AAATCTTATCTGCTTCCCTATTTGTAA
TTN-AS1	TCCTTAGGCATCACCTAGCC	GATGGAGGAAGTAGAGTCATTGG
LOC728431	CACTCCCTAACCCGTGTCC	TCCATCAAAGCAGCCAGAC
LINC00473_v1	TATGCGCGTCAGCATACTTT	TGTCCTGTGCCTCCCTGT
LINC00473_v2	TATGCGCGTCAGCATACTTT	TCTCCCAAAGCACAACGAG
FAM222A-AS1	CAACATGGAAATGGAGACCA	CTTCCGGGATCCCAGTGT
LINC00152	GAGCCACCAGCCTCTCCT	AAAAACGATCTTGCCGACAC
LINC0541471_v1	CAGATCTTCACAGCACAGTTCC	TGCTGATCCACTTTGCTTGT
LINC0541471_v2	CACCAGCCTCTCCCTGAA	TTCGATCAAGTGTGTCATAGAGC
IDI2-AS1	GTGTTAAACAAGACAACGCTGAA	AAGAGCGCTGGAAAAACCTT
SNHG15	GCAACTCCTTTGCAAGATGC	CTCAAGGAGGGACCTCAGC
ZFP91-CNTF	TTGTTCATACTTGGCGGTGA	GGCGGGCCTAATCATTTT

## Results

### Screening of lncRNAs in chemical stress responses

**Figure 1:**
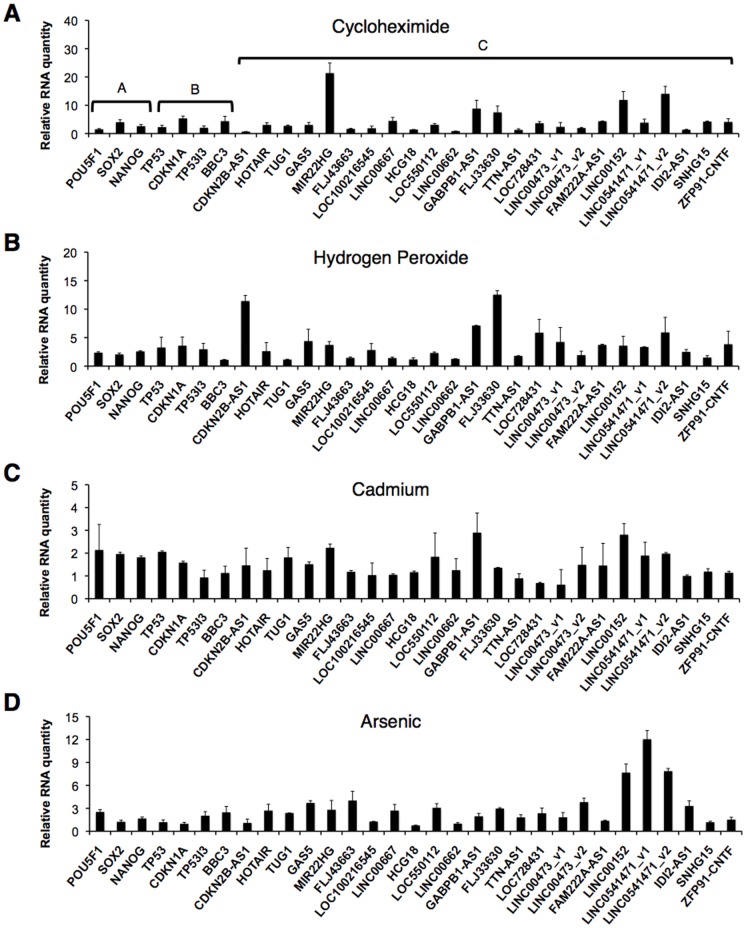
Alterations in mRNA and lncRNA expression levels by four chemical stresses in hiPSCs. Type A genes indicate pluripotency-related genes; Type B genes indicate p53-related genes; Type C genes indicate lncRNAs. hiPSCs were treated with (A) 100 µM cycloheximide, (B) 100 µM hydrogen peroxide, (C) 1 µM cadmium, or (D) 100 nM arsenic for 24 h. Expression levels of the indicated RNAs were determined by RT-qPCR. Quantitative values in response to vehicles alone were set to 1. GAPDH mRNA levels were used for normalization.

We first selected 24 lncRNAs that are short-lived (*t*_1/2_<4 h) in HeLa Tet-off cells [19], longer than 200 nt, and fulfilled the established criterion for lncRNA classification [19], [20]. Previously, we identified six lncRNAs (MIR22HG, GABPB1-AS1, FLJ33630, LINC00152, IDI2-AS1, and SNHG15) that are up-regulated by chemical stresses in HeLa Tet-off cells [20]. Recently, the expression level of LINC00152 was found to be increased in gastric carcinoma [21]. However, the biological significance of these lncRNAs is largely unknown. To investigate the responses of the 24 lncRNAs, we examined alterations in their expression levels following treatment of hiPSCs with four stresses (cycloheximide, hydrogen peroxide, cadmium, and arsenic). Cycloheximide is an inhibitor of translation, hydrogen peroxide induces oxidative stress, and cadmium and arsenic are heavy metal stresses (Fig. 1). We also investigated the responses of three pluripotency-related genes (POU5F1, SOX2, and NANOG) and four p53-related genes (TP53/p53, CDKN1A/p21, TP53I3/PIG3, and BBC3/PUMA) (Fig. 1). The p53-related genes encode proteins that respond to diverse cellular stresses.

After treatment with 100 µM cycloheximide, we found significant increases in the expression levels of MIR22HG, GABPB1-AS1, LINC00152, and LINC0541471_v2 (Fig. 1A). Treatment with 100 µM hydrogen peroxide resulted in significant increases in the expression levels of CDKN2B-AS1, GABPB1-AS1, FLJ33630, and LINC0541471_v2 (Fig. 1B). Treatment with 1 µM cadmium, there were increases in the expression levels of GABPB1-AS1 and LINC00152 (Fig. 1C). Treatment with 2.5 µM arsenic led to an increase in the expression level of LINC00152, LINC0541471_v1, and LINC0541471_v2 (Fig. 1D). In contrast, there were slightly increases (about 2-fold) in the expression levels of pluripotency-related genes by treatment with the four model stresses, but 2-fold changes is not significantly in qPCR method. This result indicated that the iPSCs were not differentiated by the model stresses at 24 h after the treatments. The expression levels of p53-related genes were changed slightly but not significantly (Fig. 1). Taken together, GABPB1-AS1, LINC00152, and LINC0541471_v2 responded to the model stresses. GABPB1-AS1 and LINC00152 responded to the model stresses in hiPSCs and HeLa Tet-off cells [20]. Therefore, these lncRNAs appear to generally and highly respond to cellular stresses. Moreover, cycloheximide and hydrogen peroxide dramatically induced these lncRNAs; thereby, we focused on cycloheximide and hydrogen peroxide in the subsequent experiments.

**Figure 2:**
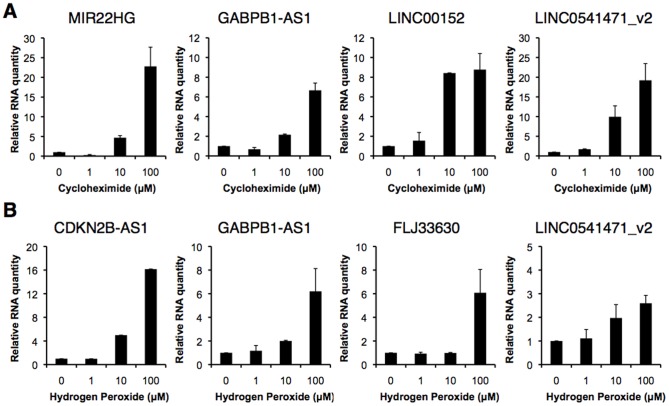
Alterations in lncRNA expression levels by two chemical stresses at various doses in hiPSCs. hiPSCs were treated with (A) cycloheximide or (B) hydrogen peroxide for 24 h. Expression levels of the indicated RNAs were determined by RT-qPCR. Quantitative values in response to vehicles alone were set to 1. GAPDH mRNA levels were used for normalization. Values represent the mean ± standard error obtained from two independent experiments.

We determined alterations in lncRNA expression levels following treatment with the two stresses (cycloheximide and hydrogen peroxide) at various doses (Fig. 2). As expected, MIR22HG, GABPB1-AS1, LINC00152, and LINC0541471_v2 levels were increased with increasing concentrations of cycloheximide (Fig. 2A). Expression levels of CDKN2B-AS1, GABPB1-AS1, FLJ33630, and LINC0541471_v2 were increased in response to increasing concentrations of hydrogen peroxide (Fig. 2B). These data indicate that these lncRNAs respond to cell stresses in a dose-dependent manner. Thus, we propose that the expression levels of these lncRNA can be used as surrogate indicators for the degrees of chemical stresses in hiPSCs.

### Novel lncRNAs highly and rapidly respond to chemical stresses

**Figure 3:**
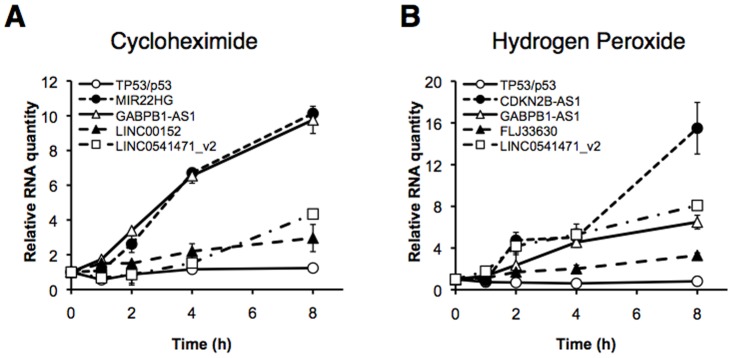
Novel lncRNAs highly and rapidly respond to chemical stresses compared with the responses of p53-related mRNAs in hiPSCs. hiPSCs were treated with (A) 100 µM cycloheximide or (B) 100 µM hydrogen peroxide. The expression levels of the indicated RNAs were determined at various time points. Quantitative values at 0 h were set to 1. GAPDH mRNA levels were used for normalization. Values represent the mean ± standard error obtained from two independent experiments.

To examine lncRNA levels and their responses to stresses in a time-dependent manner, we determined the expression levels of the lncRNAs that significantly affected by stresses at 0, 1, 2, 4, and 8 h after treatments (Fig. 3). We also investigated the response of TP53 (p53) gene as a mRNA control, which is upstream to other p53-related genes. After treatment with 100 µM cycloheximide, the expression levels of MIR22HG, GABPB1-AS1, LINC00152, and LINC0541471_v2 were higher than those of TP53 (Fig. 3A). Interestingly, MIR22HG and GABPB1-AS1 were early responders, and LINC00152 and LINC0541471_v2 were late responders. Furthermore, no dead (detached) cells were found by microscopic observation (data not shown). After treatment with 100 µM hydrogen peroxide, the expression levels of CDKN2B-AS1, GABPB1-AS1, FLJ33630, and LINC0541471_v2 were higher than those of TP53 (Fig. 3B). Interestingly, CDKN2B-AS1 and LINC0541471_v2 were early responders, and GABPB1-AS1 and FLJ33630 were late responders. Again, no dead cells were found by microscopic observation (data not shown). Compared with TP53 as a mRNA control, these data indicate that the novel lncRNAs highly and rapidly respond to chemical stresses.

## Discussion

In this study, we identified novel lncRNAs that highly and rapidly respond to general or specific stresses in hiPSCs. Using hiPSC cells, we can access to a theoretically unlimited supply of hiPSC from a diverse population. This enables to perform powerful genetic and epigenetic experiments that previously were impossible to conduct. For example, tissues like skin, peripheral blood, or other somatic tissues can be used to generate large libraries of genetically diverse iPSC lines. Such iPS libraries can be used for preclinical human trials using cell-based assays that will ideally reflect the diversity of drug responses in the population [22]. Although the functions of the identified lncRNAs remain unknown, these lncRNAs have the potential to be surrogate indicators of general or specific cellular stresses. Several lncRNAs have been identified with distinct regulatory roles in response to cellular stresses, but our present knowledge of the stress transcriptome is limited [23], [24]. Recently, two independent research groups reported that the NEAT1 lncRNA-SFPQ interaction plays roles in both repression and activation of genes, which likely depend on the context of the promoter sequence or interplay with other transcriptional factor(s) [25], [26]. Hirose et al. reported the role of NEAT1 in transcriptional regulation through sequestering of SFPQ from the RNA-specific adenosine deaminase B2 (ADARB2) gene in response to proteasome inhibition [24]. Imamura et al. reported that NEAT1 expression is induced by infection with the influenza virus or herpes simplex virus. This upregulation of NEAT1 results in relocation of SFPQ, a NEAT1-binding paraspeckle protein and repressor of *IL8* transcription, from the *IL8* promoter to the paraspeckles, leading to transcriptional activation of *IL8* [26]. In addition, most environmental stresses affect multiple signaling pathways that sense environmental conditions and coordinate various cellular activities [27]. Therefore, we believe that the relationships of the novel lncRNAs identified in this study and RNA-binding protein(s) will be elucidated in the future.
